# Repeatability of traits for characterizing feed intake patterns in dairy goats: a basis for phenotyping in the precision farming context

**DOI:** 10.1017/S1751731119002817

**Published:** 2020-05

**Authors:** S. Giger-Reverdin, C. Duvaux-Ponter, D. Sauvant, N. C. Friggens

**Affiliations:** UMR Modélisation Systémique Appliquée aux Ruminants, Inra, AgroParisTech, Université Paris-Saclay, 75005, Paris, France

**Keywords:** feeding behaviour, ruminant, intake rate, individual variability, time series

## Abstract

In ruminants, feeding behaviour variables are parameters involved in feed efficiency that show variation among individuals. This study aimed to evaluate during the first two production cycles in ruminants the repeatability of feed intake pattern, which is an important aspect of feeding behaviour. Thirty-five dairy goats from Alpine or Saanen breeds were housed in individual pens at four periods (end of first gestation, middle of first and second lactations and middle of second gestation which is also the end of first lactation) and fed a total mixed ration (**TMR**) *ad libitum*. Individual cumulative dry matter intake (**DMI**) was automatically measured every 2 min during the last 4 days of each period. Feed intake pattern was characterized by several measures related to the quantity of feed eaten or to the rate of intake during the 15 h following the afternoon feed delivery. Two main methods were used: modelling cumulative DMI evolution by an exponential model or by a segmentation-clustering method. The goat ability to sort against dietary fibre was also evaluated. There was a very good repeatability of the aggregate measures between days within a period for a given goat estimated by the day effect within breed and goat, tested on the residual variance (*P* > 0.95). The correlations between periods were the highest between the second and either the third or fourth periods. With increasing age, goats sorted more against the fibrous part of the TMR and increased their initial rate of intake. Alpine goats ate more slowly than Saanen goats but ate during a longer duration. Principal component analysis (**PCA**) was performed on all the aggregate measures of feed intake patterns. The factor score plots generated by the PCA highlighted the opposition between the different measures of feed intake patterns and the sorting behaviour. The projection of the animals on the scoring plots showed a breed effect and that there was a continuum for the feed intake pattern of goats. In conclusion, this study showed that the feed intake pattern was highly repeatable for an animal in a given period and between periods. This means that phenotyping goats in a younger age might be of interest, either to select them on feeding behaviour and choose preferentially the slow eaters or to adapt the quantity offered and restrict feed delivery to the fast eaters in order to increase feed efficiency and welfare by limiting the occurrence of acidosis, for example.

## Implications

Feeding behaviour variables are key parameters to explain some health problems in high producing ruminants when they are fed a diet of a high nutritive value to meet their requirements. However, it is known that there is a large variation among individuals. Our results show that traits characterizing feed intake patterns are repeatable for a given animal when tested at different stages during the first two production cycles but with a great variation among individuals. It might be of interest to phenotype animals in early life in order to adapt their management at a later stage and optimize feed efficiency. To do so, automatic weighing of feedstuffs could be used on farm.

## Introduction

Feeding behaviour variables are parameters involved in feed efficiency (Phocas *et al.*, 2014). However, feeding behaviour shows a high variation among individuals within an experiment (Burt, 1957; Jarrige *et al.*, 1995). It is a key factor to explain the differences in susceptibility of animals to acidosis when they receive a diet with a high proportion of concentrate to meet their requirements in intensive systems (Brown *et al.*, 2000; Desnoyers *et al.*, 2011; Gao and Oba, 2014). Indeed, there are an increasing number of automated technologies being developed, and commercialized, to track changes in animal feeding behaviour (e.g. duration and frequency of eating and ruminating bouts using neck or ear tag mounted accelerometers) for identifying at-risk animals (Thorup *et al.*, 2016). It also seems that automated measures of feed intake will soon become available for commercial use (via feed distribution systems or indirectly via, e.g., image analysis of the feed bunk). If feeding behaviour is shown to be repeatable within animal, these precision livestock farming technologies would allow very early detection of animals that are more, or less, likely to be able to cope with variable or changing diets. Accordingly, the aim of this study was, during the first two production cycles, to evaluate through different physiological stages the repeatability of feed intake pattern, which is an important aspect of feeding behaviour, and to make a first attempt to propose relevant, repeatable and easy to obtain measurements to characterize these traits in dairy goats in the context of precision livestock farming.

## Material and methods

Animals were cared for and handled in accordance with the French legislation on animal experimentation and European Convention for the Protection of Vertebrates Used for Experimental and Other Scientific Purposes (European Directive 86/609). The authorizations given by the ethical local committee (Comité d’Ethique en Expérimentation Animale, COMETHEA 45) were registered as 11-041 and 12-012.

### Animals, design, diets and feeding

Feed intake pattern was assessed in 35 dairy goats, born in early 2011, at 4 different stages, called periods (P), during the first 2 production cycles (from autumn 2011 to spring 2013):

- End of first gestation (autumn 2011, P1)
- Middle of first lactation (spring 2012, P2)
- End of first lactation and middle of second gestation (autumn 2012, P3)
- Middle of second lactation (spring 2013, P4).

During P1, goats were around the end of their second month of pregnancy (60.7 ± 10.01 days post-artificial insemination (**AI**)). During P2 and P4, they were, respectively, 75.6 ± 11.47 and 53.6 ± 7.58 days in milk (**DIM**). During P3, they were in late lactation (252.8 ± 9.98 DIM) and early pregnancy (71.3 ± 7.02 days post AI). Thus, during P2 and P4, they were at the same physiological status, but differed in age (1 *v.* 2 years old). During P1 and P3, they were also in early pregnancy and differed for 1 year in age, but during P1 they were also at a period with a significant growth rate and during P3, they were still lactating but with a lower growth rate than during P1. Therefore, age and period were confused in this trial.

All the goats were fed the same diet in the same environment during the 2-year experiment, as did their mothers fed at stall during pregnancy. The animals were all born between 4 and 17 January that was the first chosen criterion for being included in this experiment. The second criterion was a birth BW greater than 3 kg. The third one was the need to perform two lactations. Thus, among the 70 females born in 2011, only 35 were included in this study with 13 of the Alpine breed and 22 of the Saanen breed which corresponds to the ratio among the females born that year (25 Alpine and 45 Saanen goats). Kids were separated from their mothers at birth and fed good quality colostrum. Body weight was measured once in each experimental period around 1400 h before the afternoon feed delivery. During P1, mean value of BW was 49.3 (±5.68 kg, *n* = 35) with a difference between breeds: Alpine goats (45.9 ± 2.77 kg) *v.* Saanen goats (51.4 ± 5.99 kg). The goats were housed in individual pens (1.20 m by 0.75 m) during the feeding behaviour measurements, each with their own feed trough with free access to feed and water. Ten pens were fitted with weighing scales under the feed trough.

The recording lasted 12 days per goat in each period, with 8 days of adaptation to the individual pens followed by 4 days of measurements of dry matter intake (**DMI**) on 10 goats simultaneously (the goats were rotated in the stalls). During the adaptation week, the animals were housed in pens similar to those used for the measurements. Therefore, it was possible to overlap adaptation to the pen and measurements over a total duration of 4 weeks. Animals were divided into the different groups of 10 in order to obtain approximately the same physiological stage for all the goats at a given period.

Animals were fed *ad libitum* a total mixed ration (**TMR**) adapted to requirements. During P1, P3 and P4, the TMR consisted of 20% concentrate, 20% meadow hay, 30% chopped dried alfalfa (Rumiluz; Désialis, Paris, France) and 30% pressed sugar beet pulp silage on a DM basis. During P2, the TMR was slightly modified: 20% concentrate, 24% meadow hay, 27% chopped dried alfalfa and 29% pressed sugar beet pulp silage to meet lactation requirements taking into account changes in hay batch quality. The composition in ingredients and the chemical composition of the diet during the four periods are given in Table 1. Goats were machine milked twice a day in a rotary milking parlour with a low line at a *vacuum* pressure of 35 kPa, a pulsation rate of 85 pulses/min and a pulsation ratio of 65/35. Feed was delivered shortly after milking (0700 and 1500 h), with 1/3 in the morning and 2/3 in the afternoon, to take into account the time interval between milking. Diet samples and individual refusals were collected for the last 2 days of each period and were analysed separately for DM according to the International Organization for Standardization (ISO, 1983) and for cell wall components estimated by the NDF method of Van Soest and Wine (1967) modified by Giger *et al.* (1987).

**Table 1:** Ingredient composition, chemical composition and nutritive values of the diet given to 35 goats during the 4 periods (P1 to P4)

Item	P1	P2	P3	P4
Ingredients (% DM)				
Meadow hay	20	24	20	20
Chopped dried alfalfa	30	27	30	30
Pressed sugar beet pulp	30	29	30	30
Compound feed	20	20	20	20
Chemical composition (% DM)				
CP	13.0	13.1	12.7	12.0
NDF	46.5	44.8	43.9	49.5
ADF	25.1	24.0	24.8	28.0
ADL	4.4	3.6	3.9	4.9
Nutritive value				
UFL (/kg DM)	0.86	0.76	0.81	0.76
PDI (/kg DM)	65.1	67.7	63.6	64.6

UFL = net energy for lactation (unité fourragère lait); PDI = truly digestible (dietary + microbial) protein (protéine digestible dans l’intestin).

P1 is end of first gestation, P2 is middle of first lactation, P3 is end of first lactation and middle of second gestation and P4 is middle of second lactation.

### Recording and modelling of patterns of intake

Individual feed intake was automatically measured using weighing scales manufactured by Baléa SA (Saint-Mathieu-de-Tréviers, France) fitted under the feed trough (Giger-Reverdin *et al.*, 2012). This system recorded the weight of the feed contained in the trough every 2 min with a precision of 5 g. Measurements began with the afternoon feed delivery and ended 22 h later, at the removal of refusals.

Recorded data of intake of the last 4 days in each period were used to obtain cumulative DMI after each feeding. Only results obtained after the afternoon feed delivery are presented in this paper, because goats were less disturbed by the human activities on the farm during this part of the day and because they received the main part of their ration after the afternoon milking. To facilitate comparison between physiological stages and breeds, g DMI/kg BW was used in the subsequent analyses.

The time course of cumulative DMI through the 15 h from 1530 to 0630 h (450 measures) was described using the exponential model proposed by Baumont *et al.* (1990) applied to each profile (for each goat and each recording day) using the NLIN procedure of SAS (SAS, 2006):

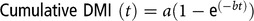

where *a* is the asymptote of the model, *b* the fractional rate of intake and *t* the time after feed delivery. The a*b product represents the initial rate of intake.

Data were also characterized by a segmentation-clustering method (Giger-Reverdin *et al.*, 2012). This method looks for changes in the slopes of cumulated feed intake and cuts the curve into segments according to their slope. The obtained segments were classified into eight clusters according to the values of their slopes: the clusters eight to six corresponded to the segments with the highest intake rates, the clusters three to five corresponded to the segments with medium intake rates and the clusters two and one corresponded to almost no intake (slope near 0).

The following aggregate measures of feed intake patterns were calculated for each goat and each recording day:

- Daily DMI (**DDMI**) or feed intake during 22 h following afternoon feed delivery
- DMI90: DMI during the 90 min following afternoon feed delivery
- DMI180: DMI during the 180 min following afternoon feed delivery
- DMI900: DMI during the 900 min (15 h) following afternoon feed delivery which corresponds to the intake between the afternoon and morning milking
- P90: proportion of feed delivery eaten during the first 90 min following afternoon feed delivery (DMI90/DMI900)
- P180: proportion of feed delivery eaten during the first 180 min following afternoon feed delivery (DMI180/DMI900)
- a: asymptote of the curve describing DMI evolution with the exponential model
- a*b: initial value of the slope of the curve describing DMI evolution with the exponential model. It corresponds to the initial rate of intake
- RMSE_ab: residual mean square error (**RMSE**) of the adjustment with the exponential model
- DMI in the 1^st^ meal after afternoon delivery: sum of the quantity of feed eaten during the first segments (from clusters eight to three) until a segment corresponding to the clusters two or one of at least 30 min was detected (Serment and Giger-Reverdin, 2012)
- NDF sorting: ratio between NDF content of intake (calculated from quantity and NDF content of offered feed and refusals) and NDF content of offered diet. When NDF sorting value <1, the goat is sorting against fibre (NDF), and when NDF sorting value ⪰1, there is no sorting against fibre (NDF) according to Leonardi and Armentano (2003).

### Statistical analysis

Factors affecting feeding behaviour variables were evaluated using the following nested model including fixed effects of breed, goat within the breed, day within breed and goat, period and the interaction between breed and period:

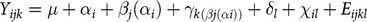


In this model, *μ* represents the overall mean, *α*_*i*_ the fixed effect of the breed (Alpine *v.* Saanen), *β*_*j*_(*α*_*i*_) the fixed effect of the goat *j* (*j* = 1 to 35) nested in the breed, *γ*_*k(βj(αi))*_ the fixed effect of the day (*k* = 1 to 4) within breed and goat, *δ*_*l*_ the period effect (*l* = 1 to 4), χ_*il*_ the interaction between breed and period and *E*_*ijkl*_ the residual error. The repeatability for a given goat within a period was estimated by the day effect within breed and goat *γ*_*k(βj(αi))*_, tested on the residual variance *E*_*ijkl.*_

When the probability of a day effect within breed and goat was higher than 0.95, which means that the repeatability of a variable within a period was high, the mean value per goat and per period was computed to test the period and the breed effects and the period*breed interaction with a simplified model. It was similar to the first model without the day effect:

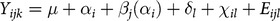


In this model, *μ* represents the overall mean, *α*_*i*_ the fixed effect of the breed (Alpine *v.* Saanen), *β*_*j*_(*α*_*i*_) the fixed effect of the goat j (j = 1 to 35) nested in the breed, *δ*_*l*_ the period effect (l = 1 to 4), χ_*il*_ the interaction between breed and period and *E*_*ijl*_ the residual error. The breed effect was tested on the goat within breed variance.

The auto-correlation between two periods was estimated by the correlation coefficients between the mean values per goat and per period.

The repeatability between periods for a given goat (correlation between repeated measures on the same animal at different periods) was estimated as the proportion of the variance between animals on the sum of the between and within animal variances (Huhtanen *et al*, 2015). It corresponds to the square of a coefficient of correlation.

A principal component analysis (**PCA**) was performed to examine the relationships among the variables. This procedure synthesizes the overall information contained in a set of observed variables into a smaller number of linear combinations of the original variables: these condensed new variables called principal components (**PC**s) are orthogonal variables. The first PC explains most of the variance, the second one most of the remaining variation and so forth. Thus, the PCA condenses the information into loadings (coefficients of the scaled variables) that show the relative importance (weighting) of the original variables in accounting for the variability in the observed data. The distribution of the observed data across the PC is shown by the scores. A statistical analysis was performed on the score plots for the first two axes of the PCA with the second simplified model: fixed effects of the breed, fixed effect of the goat nested in the breed, period effect and interaction between breed and period.

## Results

A total of 560 cumulative DMI profiles (data for 1 goat on a given day) were expected with 35 goats, 4 periods and 4 days per period. However, 19 records were either missing or discarded due to practical problems. They concerned 13 goats, within which 4 goats had 2 missing days, 1 goat had 3 missing days and 8 goats had 1 missing day. The mean value of the BW of Saanen goats measured once at the four different physiological periods was higher (*P* < 0.01) than that of Alpine goats (56.3 *v.* 51.5 kg) when tested with the second simplified model including breed, goat nested within breed and period effects and the interaction between breed and period.

### Modelling cumulative dry matter intake profile

**Figure 1:**
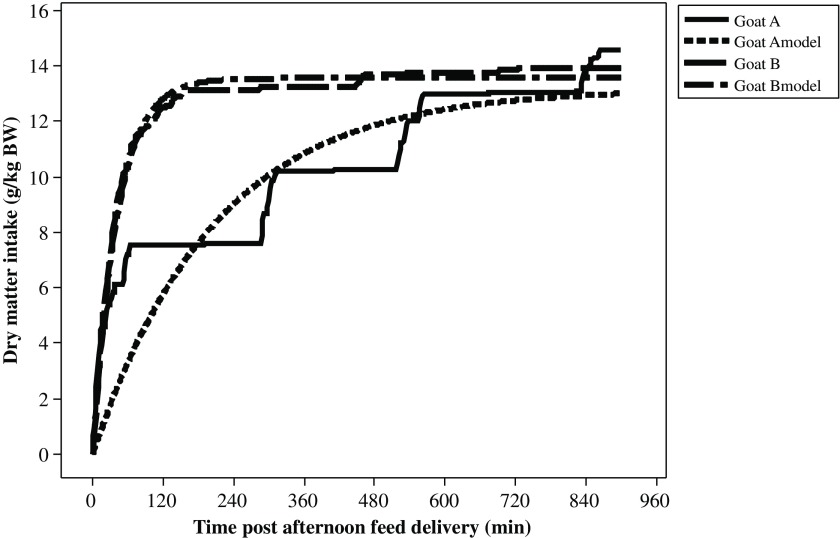
Modelling of cumulative DMI post-afternoon feeding (g/kg BW) for two goats (A and B) during the first period (end of gestation). DMI = dry matter intake.

It was possible to model all the profiles by an exponential model. Nevertheless, the RMSE of the adjusted model varied a lot, from 0.136 to 3.27 g/kg BW with a mean value of 1.32 g/kg BW and a SD of 0.514 for 541 curves. In fact, the model fitted well when the animals ate mainly during the 1^st^ meal, but not so well when the animals had several successive eating bouts as defined with the segmentation-clustering method (Figure 1). For example, for around the same DMI during the 15 h after feed delivery, the standard error was 1.51 g/kg BW for goat A and 0.285 g/kg BW for goat B measured during the first period (end of first gestation).

### Repeatability of the feed intake pattern measures between days within a period

**Figure 2:**
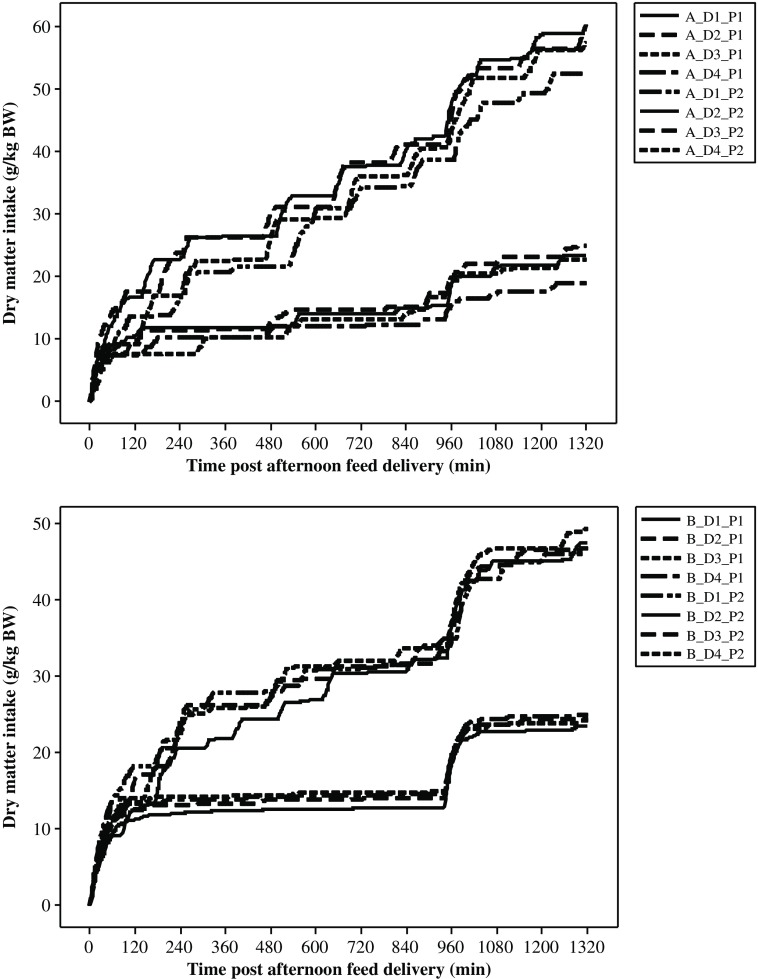
Daily cumulative DMI (g/kg BW) for goats A and B measured during 4 days (D1 to D4) in two periods (P1 end of first gestation and P2 middle of first lactation). In each figure, curves at the bottom refer to P1 and those at the top to P2. DMI = dry matter intake.

The repeatability of all aggregate measures of feed intake patterns within a period was estimated by the day effect within breed and goat, tested on the residual variance. It was high as the *P*-value was always greater than 0.95 (see Supplementary Material Table S1). Thus, there was a very good repeatability within a goat for a given period as shown, for example, in Figure 2 for two different goats. Therefore, a mean value was computed for each goat at each period averaging the daily values.

### Repeatability of the feed intake pattern measures between periods

The statistical parameters of the mean values of the aggregate measures on 35 goats during 4 periods are given in Table 2. Most of the measures had a CV around 30%. However, it was lower for NDF sorting and higher for the DMI in the 1^st^ meal. Goats sorted against the fibrous fractions of the diet despite being delivered as a TMR to limiting sorting behaviour, as NDF sorting was lower than 1. The correlations of the aggregate measures between two periods are given in Table 3. The proportions of feed eaten during either the first 90 or 180 min after feed delivery, as well as the initial slope (a*b) and the RMSE of the exponential model were highly correlated from 1 period to another. The highest correlations were between P2 and either P3 or P4: all the correlations between P2 and P3 were high (>0.60) for all the aggregate measures except NDF sorting. They remained high between P2 and P4 for DDMI and P90 and still significant (*P* > 0.05) for DMI90, P180, initial slope and RMSE of the exponential model. This means, for instance, that slow-eating goats in P2 remained slow eaters later on and fast-eating goats stayed fast eaters.

**Table 2:** Descriptive statistics of the aggregate measures of feed intake patterns for 35 goats studied across 4 different physiological stages during 4 days each

	Mean	SD	Minimum	Maximum
Bodyweight (BW kg)	54.5	7.37	39.4	73.0
Intake (g/kg BW)				
DDMI	42.5	11.55	19.6	64.4
DMI90	14.4	4.41	6.1	27.4
DMI180	19.2	6.10	7.5	35.2
DMI900	28.5	8.00	11.5	45.6
DMI in the 1^st^ meal	17.1	7.19	4.1	37.0
P90	0.519	0.1161	0.242	0.853
P180	0.682	0.1288	0.365	0.941
a	27.2	8.35	10.8	51.2
a*b	0.219	0.0960	0.058	0.516
RMSE_ab	1.32	0.453	0.28	2.80
NDF sorting	0.985	0.0162	0.943	1.030

DMI = dry matter intake; DDMI = daily DMI; DMI90 = DMI during the 90 min following afternoon feed delivery; DMI180 = DMI during the 180 min following afternoon feed delivery; DMI900 = DMI during the 900 min following afternoon feed delivery; DMI in the 1^st^ meal = sum of the quantity of feed eaten during the 1^st^ meal following afternoon feed delivery; P90 = ratio (DMI90/DMI900); P180 = ratio (DMI180/DMI900); a = asymptote of the curve describing DMI evolution with an exponential model; a*b = initial value of the slope of the curve describing DMI evolution with an exponential model; RMSE_ab = residual mean square error of the adjustment with an exponential model; NDF sorting = ratio between NDF content of intake and NDF content of offered diet.

**Table 3:** Correlation coefficients between periods (P) for the mean values per goat per period of feed intake pattern measures following afternoon feed delivery for 35 goats

	P1 to P2	P1 to P3	P1 to P4	P2 to P3	P2 to P4	P3 to P4
Intake (g/kg BW)						
DDMI	0.20	0.13	0.22	0.61**	0.46**	0.24
DMI90	0.38*	0.24	0.39*	0.60**	0.52**	0.27
DMI180	0.31	0.31	0.17	0.64**	0.31	0.21
DMI900	0.32	0.25	0.25	0.62**	0.29	0.03
DMI in the 1^st^ meal	0.48**	0.29	0.07	0.68**	0.26	0.21
P90	0.43**	0.43**	0.46**	0.59**	0.66**	0.41*
P180	0.43**	0.50**	0.28	0.59**	0.46**	0.40*
a	0.23	0.23	0.33	0.67**	0.31	0.19
a*b	0.56**	0.46**	0.41*	0.60**	0.58**	0.35*
RMSE_ab	0.58**	0.43**	0.44**	0.61**	0.55**	0.68**
NDF sorting	0.36*	0.28	0.03	0.32	0.14	0.49**

DMI = dry matter intake; DDMI = daily DMI; DMI90 = DMI during the 90 min following afternoon feed delivery; DMI180 = DMI during the 180 min following afternoon feed delivery; DMI900 = DMI during the 900 min following afternoon feed delivery; DMI in the 1^st^ meal = sum of the quantity of feed eaten during the 1^st^ meal following afternoon feed delivery; P90 = ratio (DMI90/DMI900); P180 = ratio (DMI180/DMI900); a = asymptote of the curve describing DMI evolution with an exponential model; a*b = initial value of the slope of the curve describing DMI evolution with an exponential model; RMSE_ab = residual mean square error of the adjustment with an exponential model; NDF sorting = ratio between NDF content of intake and NDF content of offered diet.

P1 is end of first gestation, P2 is middle of first lactation, P3 is end of first lactation and middle of second gestation and P4 is middle of second lactation.

* Correlations significantly different from 0; *P* < 0.05.

** Correlations significantly different from 0; *P* < 0.01.

### Effects of period and breed

The period*breed interaction observed for DMI900 and ‘a’ from the curve was linked to a significant between breeds difference at P2 (38.8 *v.* 33.8 g DMI/kg BW for the Alpine and the Saanen, respectively). At the other periods, Alpine goats ate more than Saanen, but the difference was not significant. The period effect was highly significant for all the variables (Table 4). The DDMI and the DMI900, both scaled to kg BW, were the highest during the middle of the first lactation when the animals were still growing and had a milk yield similar to the one during the last period, but higher than during the third period at the end of the first lactation. The intake after either 90 or 180 min increased with the age of the animal, possibly reflecting the increase in rumen volume and rate of intake. The amount of feed eaten in the 1^st^ meal ranked similarly to the DMI during the first 3 h after afternoon feed delivery. P90 and P180 were high in P1 and were the lowest in P2, when the animals had the greatest daily intakes. P90 and P180 returned in P4 to values close to the ones observed in P1. The ‘a’ value of the exponential model corresponded to the asymptote of the model and was very close to the DMI900 value. The initial rate of intake increased with the age of the animal. With increasing age, animals sorted more against fibre as shown by the ratio between the NDF composition of the feed eaten and the feed offered (NDF sorting) which was less than one and decreased with age. It means that the goats sorted against fibre and were more able to choose the best nutritive part of the diet when ageing.

**Table 4:** Repeatability and effect of breed and period and their interaction for the mean values per goat per period (P) of BW and feed intake pattern measures following afternoon feed delivery for 35 goats

	Repeatability	Period effect				Breed effect		*P*-value		
	*R*^2^	P1	P2	P3	P4	Alpine	Saanen	Period	Breed	Interaction
										Period*Breed
BW (kg)	0.82	48.6^A^	48.6^A^	59.3^B^	59.0^B^	51.5^a^	53.3^b^	0.00	0.00	0.59
Intake (g/kg BW)										
DDMI	0.40	25.6^A^	53.3^B^	42.2^C^	50.9^D^	44.8^a^	41.2^b^	0.00	0.00	0.07
DMI90	0.54	9.3^A^	14.5^B^	15.1^B^	18.3^C^	13.8	14.7	0.00	0.23	0.92
DMI180	0.48	11.4^A^	19.9^B^	19.8^B^	25.4^C^	18.8	19.4	0.00	0.48	0.83
DMI900	0.40	16.7^A^	36.3^B^	29.3^C^	33.1^D^	30.0^a^	27.6^b^	0.00	0.00	0.01
DMI in the 1^st^ meal	0.49	8.8^A^	16.3^B^	18.3^B^	24.4^C^	16.4^a^	17.4^b^	0.00	0.41	0.98
P90	0.56	0.563^A^	0.406^B^	0.517^A^	0.551^A^	0.474^a^	0.545^b^	0.00	0.01	0.36
P180	0.55	0.691^A^	0.552^B^	0.681^A^	0.765^C^	0.635^a^	0.709^b^	0.00	0.01	0.05
a	0.45	15.2^A^	35.8^B^	27.6^C^	31.3^D^	28.6^a^	26.3^b^	0.00	0.01	0.01
a*b	0.59	0.151^A^	0.186^B^	0.220^B^	0.297^C^	0.192	0.235	0.00	0.05	0.33
RMSE_ab	0.58	1.00^A^	1.68^A^	1.45^C^	1.34^C^	1.55	1.18	0.00	0.00	0.89
NDF sorting	0.42	0.999^A^	0.990^B^	0.980^C^	0.975^C^	0.989	0.983	0.00		0.99

DMI = dry matter intake; DDMI = daily DMI; DMI90 = DMI during the 90 min following afternoon feed delivery; DMI180 = DMI during the 180 min following afternoon feed delivery; DMI900 = DMI during the 900 min following afternoon feed delivery; DMI in the 1^st^ meal = sum of the quantity of feed eaten during the 1^st^ meal following afternoon feed delivery; P90 = ratio (DMI90/DMI900); P180 = ratio (DMI180/DMI900); a = asymptote of the curve describing DMI evolution with an exponential model; a*b = initial value of the slope of the curve describing DMI evolution with an exponential model; RMSE_ab = residual mean square error of the adjustment with an exponential model; NDF sorting = ratio between NDF content of intake and NDF content of offered diet.

P1 is end of first gestation, P2 is middle of first lactation, P3 is end of first lactation and middle of second gestation and P4 is middle of second lactation.

Repeatability between periods for a given goat was estimated as the ratio between the variance between animals and the sum of the between and within animal variance.

Within a row, ^a,b^ values with different superscripts differ significantly at *P* < 0.05 for breed effect, and ^A,B,C,D^ values differ for period effect.

There was no breed effect for the DMI in the first hours after the feed delivery, but it was significant for the DMI900, the ‘a’ parameter and the DDMI. The corresponding values were higher for the Alpine breed compared to Saanen. As a consequence, the P90 and P180 were lower for the Alpine breed compared to Saanen.

### Relationships between the feed intake pattern measures

**Figure 3:**
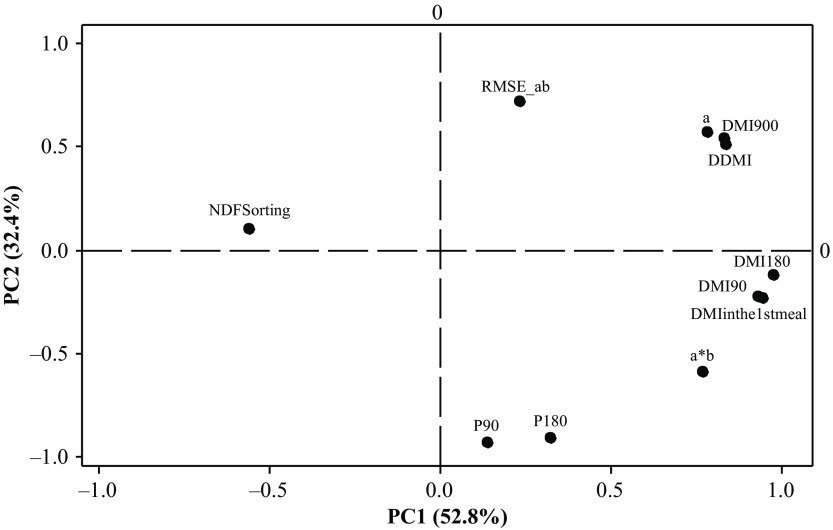
Results of a PCA based on measurements characterizing patterns of intake obtained in 35 goats during the 15 h following afternoon feed delivery presented as a loading plot of 11 variables. The data included all four measurement periods. The percentage of total variance accounted for by each of the first two PCs is shown in parentheses. PCA = principal component analysis; PC = principal component. DMI = dry matter intake; DDMI = daily DMI (g/kg BW); DMI90 = DMI during the 90 min following afternoon feed delivery (g/kg BW); DMI180 = DMI during the 180 min following afternoon feed delivery (g/kg BW); DMI900 = DMI during the 900 min following afternoon feed delivery (g/kg BW); DMI in the 1^st^meal = sum of the quantity of feed eaten during the 1^st^ meal following afternoon feed delivery (g/kg BW); P90 = ratio (DMI90/DMI900); P180 = ratio (DMI180/DMI900); a = asymptote of the curve describing DMI evolution with an exponential model; a*b = initial value of the slope of the curve describing DMI evolution with an exponential model; RMSE_ab = residual mean square error of the adjustment with an exponential model; NDF sorting = ratio between NDF content of intake and NDF content of offered diet.

A PCA was performed on the mean values per goat and per period (i.e. 35 × 4 = 140 observations) of the 11 aggregate measures previously described. Around 84% of the total variance was explained by the first two components (Figure 3). On the first axis, an opposition existed between the different expressions of DMI (DDMI, DMI90, DMI180, DMI900, DMI in the 1^st^ meal, ‘a’) and the initial intake rate of the curve describing cumulative DMI on the one hand, and NDF sorting on the other hand. This means that there was a positive relationship between the level of intake and the sorting behaviour. The goats which sorted the most against fibre (the lowest values of NDF sorting) ate more than their counterparts. On the second axis, an opposition existed between the RMSE of the exponential model, DDMI, DMI900 and ‘a’ on the one hand, and P90 or P180, and a*b to a lesser extent, on the other hand. Cumulative DMI profiles that started more quickly were therefore better fitted by an exponential model, and conversely those that started more slowly were less well fitted by the model.

**Figure 4:**
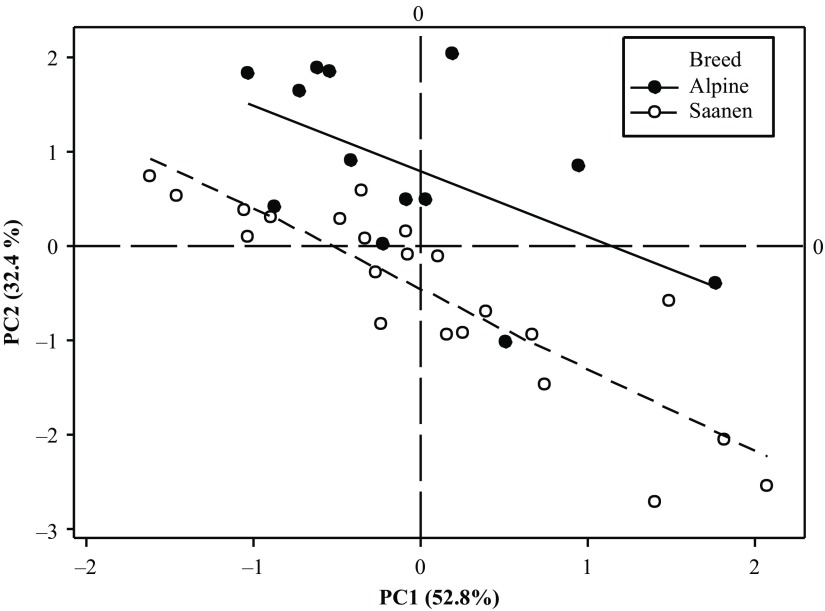
Results of a PC analysis based on aggregate measurements characterizing patterns of intake during the 15 h following afternoon feed delivery presented as score plots obtained in 35 goats at 4 different physiological stages. Means of the 4 periods are represented for each of the 35 goats. PC = principal component

According to the second simplified model of variance analysis on the score plots, the interaction between the breed and the period was never significant (*P* = 0.85 for PC1 and *P* = 0.12 for PC2). The period effect was highly significant for the 2 PC (*P* < 0.001). The breed effect was only significant for PC2 (*P* < 0.001) while the goat effect nested within breed was highly significant for both PC (*P* < 0.001). The repeatability between periods was good, as the square of the coefficient of correlation was equal to 0.54 for the scores on PC1 and of 0.59 for PC2. Given this, the barycentre of the four scoring plots per animal was computed. The projection of the animals on the scoring plot defined by the first two components showed that there was a continuum for the feed intake pattern of goats (Figure 4). Fast eaters were in the bottom right of the graph and slow eaters on the left upper part. The breed effect was significant in all periods (see Supplementary Material Figure S1).

## Discussion

As observed since a long time in cows, there is a high variation among individuals in feed intake pattern (Burt, 1957). The present study has clearly shown that there was a rather good repeatability for a given goat of its feed intake pattern across 2 years. These results were in agreement with previous ones obtained either in goats (Daovy *et al.*, 2008) or in cows (Vasilatos and Wangsness, 1980; Friggens *et al.*, 1998). In agreement with Vasilatos and Wangsness (1980), eating behaviour can be considered as a characteristic of individuals as there is a relative constancy of feeding rate of an individual in a given environment (Nielsen, 1999).

The positive relationship between the rate of intake and sorting behaviour seemed to signify that some goats were more efficient than others in their eating behaviour as they were able to sort efficiently against parts of lower nutritive value. Goats are known to be animals which sort more than other ruminants, especially than cows, due to their sharp muzzle (Morand-Fehr *et al.*, 1991; Sebata and Ndlovu, 2010). Moreover, the goats that ate a diet with a better nutritive value ate more than their counterparts. This can be explained by the notion of physical limitation of intake due to the physical fill effect of the diet (Conrad *et al.*, 1964). However, these observations were done in stall-feeding conditions with no competition between animals and no restriction in access time to the trough while sorting is a time-consuming behaviour that limits intake in some environments.

The good repeatability of eating pattern for a given goat is very interesting from a phenotyping point of view. It appears possible to phenotype an animal’s feeding behaviour in its early life, and it may thus be a relevant proxy for traits such as digestive efficiency or susceptibility to acidosis. From a practical point of view, DDMI (or intake after the afternoon feed delivery) and P90 (or P180) seemed to be the most relevant criteria to characterize eating behaviour as they were highly repeatable and can be easily measured in systems where the feed intake is automatically recorded. In the context of precision livestock farming, it can be proposed to characterize eating behaviour at the 1^st^ pregnancy to phenotype the animals and eventually adapt diets to animals.

This study also found that the residuals of the exponential model of Baumont *et al.* (1990) were greater when the animals were dividing their intake into small eating bouts and ate globally more slowly. Thus, the RMSE of the adjustment of this model seems to be a good indicator of the number of meals determined with the segmentation-clustering method. Therefore, this easy to perform method can allow to characterize the different types of cumulative DMI.

As already indicated, changes in feeding behaviour variables might be the sign of sickness (Brown-Brandl and Eigenberg, 2011). In fact, rate of intake influences rumen fermentation and the occurrence of acidosis (Desnoyers *et al.*, 2011). In a related study using the same animals, 12 of them with widely differing intake rates were switched to a high-concentrate diet. The between-goat ranking for intake rates remained the same despite the shift in diet composition to a more acidogenic one (Giger-Reverdin and Sauvant, 2016). This suggests that there may be value in testing animals when they are young in order to adapt their management later on, for example, to restrict feed access to those with the highest intake rate in order to decrease the risk of occurrence of acidosis in the herd, or to preferentially keep the animals with the lowest rates of intake.

However, part of these results need to be taken with caution before being extrapolated because they were obtained on a set of 35 goats without any competition for access to the feed trough which might decrease DMI or increase the feeding rate (Campling and Morgan, 1981; Hosseinkhani *et al.*, 2008). They must also be confirmed on a larger set of data with animals fed in groups.

The increase in the rate of intake as goats get older is in agreement with previous trials on cows: primiparous cows ate more slowly than multiparous ones (Burt, 1957; Beauchemin and Rode, 1994; Neave *et al.*, 2017). Moreover, lactating animals tend to eat more than dry ones as their requirements are higher (Campling and Morgan, 1981).

Even after scaling to BW, Alpine goats ate more than Saanen but ate at a lower speed. They ate during a longer duration or had more eating bouts as estimated by the higher standard error of the exponential model describing feed intake evolution. It may be speculated that Alpine goats had consequently more steady-state conditions in the rumen than Saanen and a better rumen digestion as organic matter digestibility of Alpine goats was 2.2 points higher than Saanen ones with the same acidogenic diet (Giger-Reverdin and Sauvant, 2016). Differences between breeds were also observed on eating patterns in cows (Dürst *et al.*, 1993; Senn *et al.*, 1995).

Mechanistic models of intake and digestion are already able to predict the average DMI profile for a given type of diet either indoors (Sauvant *et al.*, 1996) or during grazing (Baumont *et al.*, 2004). It has also been shown by Giger-Reverdin *et al.* (2010) that differences in intake rate which impacted rumen pH responses can be fairly simply and mechanistically modelled. The present results suggest that such models could be deployed in conjunction with precision livestock measures of feeding behaviour for large-scale phenotyping of digestive efficiency.

## Conclusion

This study showed that even if a high between-animal variation was observed in feed intake patterns, there was a high repeatability of most of the studied behavioural variables within a period and a good repeatability of feed intake pattern for a given animal when tested at different stages during the first two production cycles. The most repeatable measures were the percentages of feed eaten during the first hours after feed delivery, which were independent of the level of intake. These findings suggest that it could be of interest to phenotype all the goats in a herd for their feed intake pattern in early life in order either to select the slow eaters or to restrict feed delivery to the fast eaters in order to increase feed efficiency and welfare by limiting the occurrence of acidosis, for example. On farm automatic weighing devices could be used for this purpose.

## Software and data repository resources

None of the data were deposited in an official repository.

## Supplementary material

For supplementary material accompanying this paper visit http://dx.doi.org/10.1017/S1751731119002817. click here to view supplementary material
