# Supplementary material for: Repeatability of traits for characterizing feed intake patterns in dairy goats: a basis for phenotyping in the precision farming context
Source: Animal. 2019 Nov 26;14(5):1083–92. doi: 10.1017/S1751731119002817 (PMC7163394; doi:10.1017/S1751731119002817)
Supplement: Supplementary file 1 [file S1751731119002817sup.zip › S1751731119002817sup001.docx]

Animal Journal

# Supplementary material.

# Repeatability of traits for characterizing feed intake patterns in dairy goats: a basis for phenotyping in the precision farming context

# S. Giger-Reverdin, C. Duvaux-Ponter, D. Sauvant and N. C. Friggens

Figure S1 Results of a principal component (PC) analysis based on aggregate measurements characterizing patterns of intake during the 15 hours following afternoon feed delivery presented as score plots obtained in 35 goats at four different physiological stages. Each period is shown on a separate graph
