# Supplementary material for: Repeatability of traits for characterizing feed intake patterns in dairy goats: a basis for phenotyping in the precision farming context
Source: Animal. 2019 Nov 26;14(5):1083–92. doi: 10.1017/S1751731119002817 (PMC7163394; doi:10.1017/S1751731119002817)
Supplement: Supplementary file 1 [file S1751731119002817sup.zip › S1751731119002817sup002.docx]

Animal Journal

# Supplementary material. Repeatability of traits for characterizing feed intake patterns in dairy goats: a basis for phenotyping in the precision farming context

S. Giger-Reverdin, C. Duvaux-Ponter, D. Sauvant and N. C. Friggens

# Table S1

# Analysis of the repeatability of the feed intake pattern measures between days within a period for 35 goats

|  | Mean square values | | | | | |  | |
| --- | --- | --- | --- | --- | --- | --- | --- | --- |
|  | Period | Breed | Goat (Breed) | Day(Breed Goat) | Period*Breed | Residual variance | Day effect | |
|  |  |  |  |  |  |  | F value | P value |
| DF | 3 | 1 | 33 | 105 | 3 | 395 |  |  |
| Item |  |  |  |  |  |  |  |  |
| BW (kg) | 4575 | 2941 | 270 | 0.10 | 11.83 | 5.33 | 0.019 | 1.00 |
| DDMI (g/kgBW) | 18715 | 1073 | 82.9 | 7.41 | 127 | 15.3 | 0.48 | 1.00 |
| DMI90 | 1727 | 116 | 72.6 | 2.65 | 3.41 | 7.44 | 0.36 | 1.00 |
| DMI180 | 4088 | 57.5 | 95.14 | 3.78 | 9.74 | 11.09 | 0.34 | 1.00 |
| DMI900 | 9424.26 | 731.0 | 45.6 | 3.02 | 99.9 | 7.36 | 0.41 | 1.00 |
| DMI in the 1stmeal | 5049 | 129 | 161 | 10.5 | 5.61 | 21.09 | 0.50 | 1.00 |
| P90 | 0.647 | 0.644 | 0.0753 | 0.00274 | 0.0204 | 0.007227 | 0.38 | 1.00 |
| P180 | 0.947 | 0.715 | 0.082 | 0.0039 | 0.0559 | 0.0085 | 0.46 | 1.00 |
| a | 9990 | 678 | 75.8 | 2.97 | 140 | 9.52 | 0.31 | 1.00 |
| a*b | 0.471 | 0.232 | 0.057 | 0.0015 | 0.0026 | 0.0044 | 0.33 | 1.00 |
| RMSE_ab | 10.5 | 16.7 | 1.05 | 0.093 | 0.256 | 0.120 | 0.77 | 1.00 |
| NDFSorting | 0.00735 | 0.00168 | 0.00056 | 0.000138 | 0.000026 | 0.00019 | 0.72 | 1.00 |

DF: degrees of freedom

The repeatability for a given goat within a period was estimated by the day effect within breed and goat, tested on the residual variance

DMI =dry matter intake, DDMI = daily DMI, DMI90 = DMI during the 90 minutes following afternoon feed delivery, DMI180 = DMI during the 180 minutes following afternoon feed delivery, DMI900 = DMI during the 900 minutes following afternoon feed delivery, DMI in the 1^st^meal = sum of the quantity of feed eaten during the 1^st^ meal following afternoon feed delivery, P90 = ratio (DMI90/DMI900), P180 = ratio (DMI180/DMI900), a = asymptote of the curve describing DMI evolution with an exponential model, a*b = initial value of the slope of the curve describing DMI evolution with an exponential model. RMSE_ab = residual mean square error of the adjustment with an exponential model, NDFSorting = ratio between NDF content of intake and NDF content of offered diet.
